# Association of Patient-Centered Medical Home designation and quality indicators within HRSA-funded community health center delivery sites

**DOI:** 10.1186/s12913-020-05826-x

**Published:** 2020-10-27

**Authors:** Nathaniel Bell, Rebecca Wilkerson, Kathy Mayfield-Smith, Ana Lòpez-De Fede

**Affiliations:** 1 College of Nursing, University of South Carolina, Columbia, USA; 2 Division of Integrated Health and Policy Research, Institute for Families in Society, University of South Carolina, Columbia, USA; 3 Division of Integrated Health and Policy Research, Institute for Families in Society, University of South Carolina, 1600 Hampton St., Suite 507, Columbia, SC 29208 USA

**Keywords:** Health Resources and Services Administration, Community Health centers, Medical home, Geographic mapping

## Abstract

**Background:**

Patient-Centered Medical Home (PCMH) adoption is an important strategy to help improve primary care quality within Health Resources and Service Administration (HRSA) community health centers (CHC), but evidence of its effect thus far remains mixed. A limitation of previous evaluations has been the inability to account for the proportion of CHC delivery sites that are designated medical homes.

**Methods:**

Retrospective cross-sectional study using HRSA Uniform Data System (UDS) and certification files from the National Committee for Quality Assurance (NCQA) and the Joint Commission (JC). Datasets were linked through geocoding and an approximate string-matching algorithm. Predicted probability scores were regressed onto 11 clinical performance measures using 10% increments in site-level designation using beta logistic regression.

**Results:**

The geocoding and approximate string-matching algorithm identified 2615 of the 6851 (41.8%) delivery sites included in the analyses as having been designated through the NCQA and/or JC. In total, 74.7% (*n* = 777) of the 1039 CHCs that met the inclusion criteria for the analysis managed at least one NCQA- and/or JC-designated site. A proportional increase in site-level designation showed a positive association with adherence scores for the majority of all indicators, but primarily among CHCs that designated at least 50% of its delivery sites. Once this threshold was achieved, there was a stepwise percentage point increase in adherence scores, ranging from 1.9 to 11.8% improvement, depending on the measure.

**Conclusion:**

Geocoding and approximate string-matching techniques offer a more reliable and nuanced approach for monitoring the association between site-level PCMH designation and clinical performance within HRSA’s CHC delivery sites. Our findings suggest that transformation does in fact matter, but that it may not appear until half of the delivery sites become designated. There also appears to be a continued stepwise increase in adherence scores once this threshold is achieved.

## Background

One of the primary tenets of the Affordable Care Act (ACA) was to reform health care delivery through funding for patient-centered care. Its impact on primary care has been far-reaching, particularly the ACA-related patient-centered medical home (PCMH) initiative [1–3]. The underlying intent of PCMH-modeled care was to transform a fragmented primary care system historically oriented toward volume over value into a patient-centered, comprehensive, coordinated, and accessible health care delivery model that is committed to quality and safety [4–7]. Over the last decade, both the public and private payers have experimented with various PCMH payment approaches and incentives of various kinds that link how much physicians are paid to how well they perform on dimensions of cost or quality [8].

Despite substantial growth in the number medical homes [9, 10], overall reviews of practice transformation continue to yield inconsistent findings [4, 11–13]. Many see the driving force behind variation in PCMH outcomes as stemming from differences in their design and implementation [13]. For example, some homes may choose to focus on better care coordination, others may prioritize improved patient tracking, whereas others may emphasize access. Nor are all medical homes equal. Their processes and outcomes are influenced by the practice size, location (e.g., urban versus rural), patient mix, years of recognition, as well as ownership [13–17].

Variation in PCMH implementation has similarly been found within Health Resources and Service Administration (HRSA) community health center (CHC) evaluations [18–24]. CHCs are funded through HRSA to provide primary care services to over 23 million persons, the vast majority of whom are among the country’s most socially and geographically vulnerable [25]. As of 2018, three quarters of HRSA’s 1352 CHCs were designated medical home practices (i.e., accredited/certified), making HRSA one of the foremost adopters of medical home-modeled care throughout the country [26].

A well-known critique unique to HRSA’s PCMH evaluations is that it is not possible to determine the degree in which its transition is impacting performance [18, 27]. At issue is that HRSA reports its PCMH clinical performance scores at the CHC level, but a CHC’s performance is based on data submitted from its delivery sites. A single CHC can oversee dozens of delivery sites, many or potentially the majority of which may not be designated medical homes. Moreover, in most cases, services delivered by the grantee (i.e., CHC) that become part of its performance report are provided on-site at one of its service delivery locations [28]. Similar limitations emerged from the 2006 and 2009 Commonwealth Fund surveys of CHC providers [23, 24]. Besides lack of currency, the Commonwealth surveys perpetuate limitations of ecological fallacy because PCMH designation was defined as a CHC that managed ‘at least one’ certified site. Both limitations are problematic for determining whether delivery sites that choose to embrace PCMH principles and apply for designation, or those that choose not to seek it, matters. In particular, factors associated with the designation of a single clinic may not be associated with clinical care across all delivery sites. The lack of differentiation between site-level designation versus organizational-level recognition is seen as a potential reason for the lack of consistency linking PCMH designation to improvements in clinical performance [18].

The purpose of this study was to examine whether the proportion of designated delivery sites within a CHC’s network is associated with improved clinical performance scores. Our aim was to determine whether there was a stepwise increase in performance as the proportion of a CHCs designated delivery sites increased. To our understanding, this is the first attempt to address a long-standing concern as to the use of CHC-level data to capture the true extent that PCMH principles have been implemented. Accordingly, this study sets out to answer the following research question: is there a positive association between clinical performance and PCMH status as the proportion of a CHC’s designated delivery sites increases? To answer this question, we developed a sustainable and transparent approach for using geocoding and approximate string-matching algorithms to merge PCMH site-level designation data with the HRSA uniform data system (i.e., UDS) clinical reports and analyze the association between clinical quality and PCMH designation using publicly available data.

## Methods

### CHC and delivery site spatial data

All CHC and its delivery site spatial and attribute data were obtained through HRSA’s ArcGIS server connection (https://gisportal.hrsa.gov/server/rest/). Delivery site identifiers were determined from the ‘Primary Health Care Facility’ spatial layer. All CHCs were identified from its attribute table. Specific queries using variations of the search terms ‘dental’ and ‘eye’ and ‘urgent care’ were run in order to assess whether delivery sites other than primary care practices were identified. No positive matches were found. We excluded all Alaskan, Hawaiian, and other overseas delivery sites due to spatial data that we had available for geocoding. We also excluded clinics designated as mobile units, seasonal facilities, and those designated only to migrant populations. Mobile sites were excluded as they are not restricted to a serving a particular location. Seasonal and migrant facilities were excluded as they often have different funding parameters than other delivery sites. These exclusions reduced the number of potentially eligible CHCs from 1457 to 1387 and the number of potentially eligible delivery sites from 12,549 to 8670.

### PCMH identification

HRSA contracts with the Accreditation Association for Ambulatory Health Care (AAAHC), the Joint Commission (JC), and the National Committee for Quality Assurance (NCQA) to provide technical assistance and training for CHCs that elect to begin the PCMH designation processes. We limited our analysis to delivery sites that had obtained recognition through the NCQA and/or JC as both organizations require site-level certification. We excluded the AAAHC-designated sites from the analysis as it uses a network accreditation process, which would bias both the evaluation of the geocoding methodology as well as our objective to monitor an incremental increase from designation status on changes in quality measure scores. The NCQA is the predominant accrediting body through which CHCs seek certification. As this study was limited to publicly available reports, NCQA recognition was defined using its July 2018 roster whereas JC-accredited sites were identified from its April 2018 report. We use the term ‘designated’ medical home throughout the remainder of this paper to refer to a site that met the accreditation/certification requirements to obtain PCMH status.

### Data linkages and address mapping

Our initial linkage employed a composite geocoding methodology. We linked address information (e.g., street name, street suffix, ZIP code) listed in the UDS, NCQA, and the JC to street centerline data using the ESRI Street Map Premium Address File, which is an enhanced version of commercial street reference data from HERE, TomTom, and INCREMENT P. Prior to geocoding, we standardized each address file to US Postal Service mailing format to increase the likelihood of matching the provider address information with the street centerline file. Standardization was done using ZP4 address correction software.

At issue, however, is that HRSA, NCQA, and JC all use different legal and shorthand naming and address conventions to refer to identical facilities (e.g., MedLink Gainesville vs. MedLink, Inc. - Gainesville). We developed an approximate string algorithm using SQL in order to avoid dropping joins that would otherwise fail due to naming inconsistencies. Three different conditions were written in order to link delivery sites. All conditions required a name similarity tolerance. Condition 1 required an exact match on the street, city, state, and with a name similarity tolerance value greater than 0.30 (range 0.0–1.0). Condition 2 required an exact match for the street and state and name similarity tolerance, but allowed the city name to have a less than perfect match. Condition 3 required a perfect match on the city and state, a less than perfect match on the street, an exact match on the street name suffix (e.g., Suite #), and an address similarity of 0.90. All algorithms required an exact match on ZIP code. Over 90% of all linkages were obtained using the matching Condition 1. Our name similarity tolerance of 0.30 was derived from manual observation of data linkages and by examining the match information in every 20th record.

### HRSA clinical performance measures

All quality measures were abstracted from HRSA’s 2018 UDS national clinical quality reports [29]. The UDS releases clinical, cost, patient, and programmatic requirement data for each CHC on an annual basis. Variations of these clinical end points are also routinely collected by the NCQA through its Healthcare Effectiveness Data and Information Set (HEDIS) program [30]. Clinical measures were grouped into 3 types: perinatal health, preventive health screening and services, and chronic disease management, including: (1) access to prenatal care during the first trimester, (2) low birth weight, (3) cervical cancer screening, (4) weight assessment and counseling for nutrition and physical activity for children and adolescents, (5) weight assessment and counseling for nutrition and physical activity for adults, (6) colorectal cancer screening, (7) childhood immunizations, (8) use of appropriate medications for asthma, (9) coronary artery disease (CAD) lipid therapy, (10) ischemic vascular disease (IVD) use of aspirin or another antithrombotic, and (11) controlling high blood pressure among hypertensive patients with blood pressure < 140/90).

### Covariates

We used patient demographic and facility characteristics released in the UDS to identify potential control variables. Patient characteristics included child, adult, and elderly adult age distributions; the percent minority; living below the poverty line; insurance type; and co-morbidity measured using hypertension and diabetes rates. Institution characteristics included urban clinic location, total costs per patient, total number of delivery sites, and average patient size.

### Analysis

We used a beta logistic regression model using maximum likelihood estimation to predict the adjusted effects of an increase in site-level PCMH designation on changes to a CHC’s clinical performance scores. A logit link was used to ensure that the confidence intervals for the predicted mean were between the bounds of (0,1). To avoid data loss, we amended less than 1% of the clinical performance scores to fall between the proportions of 0 and 1. Based on the premise that PCMH designation should improve overall patient care, we hypothesized that an increase in the number of designated sites would positively correlate with an increase in a CHC’s adherence rates for each process and outcome measure, presuming similarities in design with respect to the clinical, demographic, and geographic conditions previously found to confound this association [14–17].

Additional contrasts were conducted to assess whether discrete changes (i.e., 10%) in the proportion of a CHC’s designated sites resulted in a stepwise increase in its adherence scores. As the data reported by HRSA are already published as percentages, this allowed us to model percentage point differences in clinical performance against discrete increases in PCMH saturation. In this manner, the difference in adherence rates by percentile group can be assessed against specific designation targets (e.g., designating 50% of all sites) that policy makers might use.

All marginal effects were contrasted against the predicted probability scores among CHCs that had no designated delivery sites. Patient and clinical characteristics with statistically significant (*p* < 0.20) differences across PCMH saturation groups were included as covariates in the regression models. All models were adjusted for the number of delivery sites that each CHC managed. We excluded all false positive and negative matches from the regression models in order to minimize the likelihood of inflating/deflating comparisons owing to errors in the geocoding algorithm. We determined whether the geocoding algorithm resulted in a false positive/negative match at the CHC level by contrasting the list of identified sites against HRSA’s PCMH recognition initiative database [26]. If a CHC was not listed on HRSA’s PCMH recognition database then the match was determined to be inaccurate. Point estimates for sensitivity, specificity, false positive probability, and false negative probability were identified using cross tabulation and Chi-Square tests. All statistical analyses were conducted using SAS, version 9.4 for Windows.

## Results

Table 1 and shows the total number of CHCs and the proportion of delivery sites included in the analysis prior to the removal of false positive/negative matches. In total, the data initially consisted of 1239 CHCs overseeing 8095 delivery sites that were identified through linkages with UDS clinical and demographic data. The geocoding and string-matching algorithm resulted in 755 true positive and 242 true negative CHC matches, in addition to 38 false positive and 204 false negative matches, for an overall accuracy rate of 80%, with a positive predictive value of 79% and a negative predictive value of 86%. We had to exclude 42 of the 1281 records from the sensitivity analysis because the CHC listed in HRSA’s recognition initiative file was not listed in its UDS database. False negative rates were less than 10% within 22 states and the District of Columbia, and under 20% within 38 states overall. Two states, Oregon and Minnesota, accounted for 39 of the 204 (19%) false positive matches and an 87% drop rate due to naming/address discrepancies between the NCQA/JC and the UDS databases. These limitations could not be fixed using the approximate string-matching algorithm.

**Table 1:** Accuracy of the geocoding and approximate string-matching algorithm

	HRSA PCMH file (yes, PCMH)	HRSA PCMH file (no, PCMH)
Geocoding method (yes, PCMH)	755	38
Geocoding method (no, PCMH)	204	242

Overall, the matching algorithm identified 2615 of the 6851 (41.8%) delivery sites included in the final analyses as having been designated through the NCQA and/or JC. There were 157 (6.0%) delivery sites that received both NCQA and JC recognition. In total, 74.7% (*n* = 777) of the 1039 CHCs included in the analysis after removing false positive/negative matches managed at least one NCQA- and/or JC-designated site. This is slightly less than the 77% HRSA published for the same year for the lower 48 states and District of Columbia [31].

Differences in patient and clinical site characteristics among CHCs that did and did not manage at least one designated delivery site are shown in Table 2**.** CHCs that managed at least one designated site oversaw more sites (7.4 vs 4.3, *p* < 0.0001), a higher average patient load (27,212 vs. 11,329, *p* < 0.0001), as well as more sites in urban areas (40.8% vs 31.0%, p 0.006) on average than CHCs that did not manage at least one designated site. CHCs without a designated delivery site treated a higher proportions of adult patients (66.5% vs 61.2%, *p* < 0.0001) as well as different case mixes with respect to uninsured patients (28.2 vs 23.0, *p* < 0.0001) and Medicaid/CHIP (40.2% vs. 44.8%) on average.

**Table 2:** Patient and institutional characteristics within CHCs by PCMH designation status

	PCMH designation status		
Patient and facility characteristics	No designated Sites	With designated sites	***p*** value
**CHC characteristics**			
Mean number of delivery sites (SD)	4.3 (4.2)	7.4 (7.0)	< 0.0001
Urban	82 (31.0)	317 (40.8)	0.006
Total average patients (SD)	11,329 (13,047)	27,212 (29,720)	< 0.0001
Total costs per patient ($)	1006 (746)	1048 (591)	0.970
**Patient case mix characteristics**			
Children (under age 18)	23.6	28.3	< 0.0001
Adult (18–64)	66.5	61.2	< 0.0001
Older adults (age 65 and over)	10.5	10.0	0.267
Minority	56.2	54.9	0.582
Below 200% poverty line	90.1	89.6	0.527
Uninsured	28.2	23.0	< 0.0001
Medicaid/CHIP	40.2	44.8	0.001
Medicare	11.6	11.5	0.742
Hypertensive	28.8	29.3	0.478
Diabetic	15.7	15.5	0.601

All values in the table are percentages, unless noted with a ‘(SD)’ or ‘$’

Table 3 displays mean adherence scores for the perinatal, preventative, and chronic disease management measures as well as the proportion of CHCs that met or exceeded its performance target. With the exception of low birth weight and childhood immunization indicators, CHCs that managed at least one designated site had statistically significantly higher performance clinical performance scores, on average. When stratified by HRSA clinical targets for each measure, CHCs with designated delivery sites similarly outperformed non-designated sites on the same nine measures. On average, adherence targets for perinatal measures were more often met among non-PCMH CHCs, but were 11 to 14% higher among CHCs that managed designated sites for the preventative chronic disease indicators..

**Table 3:** HRSA clinical performance measure scores among CHCs with and without at least one designated delivery site

	Percentage score			No. of CHCs that met/exceeded benchmark (%)		
	No designated sites	With designated sites	***p*** value	No designated sites	With designated sites	***p*** value
**Perinatal health**						
Access to prenatal care (≥ 88.1%)^a^	61.1	68.0	0.001	22 (8.4)	107 (13.8)	0.023
Low birth weight (≤ 6.8%)^a^	10.1	9.0	0.119	154 (58.8)	362 (46.6)	< 0.0001
**Preventive health screening & services**						
Cervical cancer screening (≥ 55.7%)	45.3	53.5	< 0.0001	70 (26.7)	370 (47.6)	< 0.0001
Weight assessment & counseling (children) (≥ 65.9%)	56.1	67.1	< 0.0001	110 (42.0)	444 (57.1)	< 0.0001
Weight assessment & counseling (adults) (≥ 63.9%)	63.9	70.0	0.000	142 (54.2)	486 (62.6)	0.017
Colorectal cancer screening (≥ 42.0%)	35.8	43.6	< 0.0001	89 (34.0)	405 (52.1)	< 0.0001
Childhood immunization status (≥ 80%)^a^	32.2	34.7	0.145	15 (5.7)	14 (1.8)	0.001
**Chronic disease management**						
Use of appropriate medications for asthma (≥ 86.6%)	72.8	83.2	< 0.0001	109 (41.6)	442 (56.9)	< 0.0001
Coronary artery disease: lipid therapy (≥ 80.7%)	73.1	80.4	< 0.0001	129 (49.2)	451 (58.0)	0.013
IVD: use of aspirin or another antithrombotic (≥ 79.3%)	72.3	81.0	< 0.0001	125 (47.7)	506 (65.1)	< 0.0001
Controlling high blood pressure (hypertensive) (≥ 61.2%)^a^	58.9	63.2	< 0.0001	119 (45.4)	466 (60.0)	< 0.0001

^a^Indicates a Healthy People 2020 Benchmark

Note: HRSA performance benchmarks are shown in parentheses

We next examined adjusted odds ratios for each continuous dependent clinical performance score, a continuous independent variable of the proportion of designated delivery sites, and other covariates that were statistically significant across PCMH designation categories (see Table 4). Odds ratios less than 1 for the low birth weight indicator represent desirable effects. After adjustment for patient and clinical covariates, a one-unit change PCMH proportion increased the log odds among eight of the 11 performance measures, ranging from a 13% odds increase (OR 1.13; 95% CI 1.02–1.24) in lipid therapy adherence to a 39% odds increase (OR 1.39, 95% CI 1.23–1.57) in adherence scores for weight assessment and therapy treatment among children. Differences in odds for both prenatal indicators as well as childhood immunization adherence were mixed after adjusting for designation status.

**Table 4:** Multiple regression association between PCMH saturation within CHC delivery sites and HRSA clinical performance scores

	Access to prenatal care	Low birth weight	Cervical cancer screening	Weight assessment & counseling (children)	Weight assessment & counseling (adults)	Colorectal cancer screening	Childhood immunization status	Appropriate medications for asthma	Coronary artery disease: lipid therapy	IVD: use of aspirin or another antithrombotic	Controlling high blood pressure
Proportion of designated delivery sites	1.02 (0.88–1.19)	0.96 (0.82–1.12)	1.16 (1.06–1.26)*	1.39 (1.23–1.57)*	1.20 (1.07–1.35)*	1.22 (1.10–1.36)*	1.01 (0.86–1.18)	1.18 (1.05–1.33)*	1.13 (1.02–1.24)*	1.15 (1.05–1.26)*	1.11 (1.03–1.19)*
Number of delivery sites	0.99 (0.98–1.00)	0.99 (0.99–1.00)	0.99 (0.98–0.99)	1.00 (0.99–1.01)	1.00 (1.00–1.01)	0.99 (0.98–1.00)	0.99 (0.98–1.00)	0.99 (0.98–1.00)	0.99 (0.98–0.99)*	1.00 (0.99–1.00)	0.99 (0.99–1.00)
% child case mix	1.19 (0.76–1.86)	1.49 (0.94–2.36)	1.25 (0.95–1.63)	2.16 (1.50–3.11)*	1.32 (0.93–1.87)	1.12 (0.81–1.56)	1.81 (1.14–2.87)*	1.03 (0.73–1.47)	0.66 (0.48–0.89)*	1.20 (0.91–1.59)	0.91 (0.74–1.13)
Urban	1.06 (0.96–1.18)	1.11 (0.99–1.23)	1.04 (0.97–1.10)	0.94 (0.87–1.03)	0.95 (0.87–1.03)	1.01 (0.93–1.09)	1.14 (1.03–1.27)*	1.04 (0.96–1.12)	1.03 (0.96–1.10)	1.02 (0.96–1.09)	0.98 (0.93–1.03)
Medicaid/CHIP	1.24 (0.88–1.75)	1.01 (0.69–1.47)	1.33 (1.08–1.65)	1.29 (0.96–1.73)	1.08 (0.82–1.42)	0.67 (0.52–0.86)*	1.05 (0.72–1.53)	0.97 (0.74–1.28)	0.89 (0.70–1.12)	0.72 (0.58–0.89)*	0.90 (0.76–1.06)
No insurance	0.77 (0.54–1.11)	1.00 (0.69–1.46)	1.23 (0.99–1.52)	1.26 (0.94–1.69)	1.28 (0.98–1.68)	0.67 (0.52–0.86)	1.18 (0.81–1.72)	0.88 (0.67–1.16)	0.98 (0.79–1.23)	0.68 (0.55–0.84)*	0.63 (0.53–0.74)*
Average number of patients	1.29 (1.20–1.38)*	1.13 (1.05–1.22)*	1.18 (1.13–1.23)*	1.09 (1.03–1.15)*	1.01 (0.96–1.07)	0.67 (0.52–0.86)	1.30 (1.21–1.40)*	1.21 (1.15–1.28)*	1.21 (1.16–1.27)*	1.11 (1.07–1.16)*	1.06 (1.03–1.10)*

* *p* < 0.05

Adjusted marginal effects for discrete increases in site-level designation in 10% intervals are shown in Table 5. All changes in effects are measured as percentage point increases. All comparisons (*p* < 0.05) are in reference to CHCs that had no designated delivery sites. There were no statistically significant increases in mean predicted probability scores for the prenatal indicators as the proportion of designated medical homes increased. The predicted probability scores for access to prenatal care ranged from 58.1 to 59.0% across all intervals whereas low birth weight scores ranged from 9.6 to 9.2%. Among the preventative health and screening services measures, the strongest indicator of a proportional effect of PCMH designation was for childhood weight assessment and counseling. CHCs that designated at least 20% of its delivery sites showed a statistically significant 3.5% percentage point increase in mean predicted probability scores, rising from 57.6 to 61.2%. These changes rose stepwise to a 11.8 percentage point increase once a CHC designated at least 90% of its delivery sites. With the exception of childhood immunization scores, all other preventative health and screening scores showed that CHCs that designated at least half of its delivery sites saw statistically significant increases in mean adherence scores, ranging from a 3.0 percentage point increase in colorectal cancer screening rates to 6.7 percentage point increase in weight assessment and screening among adults.

**Table 5:** Change in marginal effects (percentage points) by discrete increases in the proportion of designated delivery sites

	0%*	1–10%	11–20%	31–30%	31–40%	41–50%	51–60%	61–70%	71–80%	81–90%	91–100%
**Perinatal health**											
Access to prenatal care	0.581	0.582 (+ 0.001)	0.583 (+ 0.002)	0.584 (+ 0.002)	0.585 (+ 0.003)	0.586 (+ 0.004)	0.586 (+ 0.005)	0.587 (+ 0.006)	0.588 (+ 0.007)	0.589 (+ 0.007)	0.590 (+ 0.008)
Low birth weight	0.096	0.096 (+ 0.000)	0.095 (−0.001)	0.095 (−0.001)	0.094 (− 0.001)	0.094 (− 0.002)	0.094 (− 0.002)	0.093 (− 0.003)	0.093 (− 0.003)	0.093 (− 0.003)	0.092 (− 0.004)
**Preventive health screening & services**											
Cervical cancer screening	0.492	0.498 (+ 0.005)	0.503 (+ 0.010)	0.508 (+ 0.015)	0.513 (+ 0.020)	0.518 (+ 0.025)	0.523 (+ 0.030)*	0.528 (+ 0.035)*	0.533 (+ 0.040)*	0.538 (+ 0.045)*	0.543 (+ 0.050)*
Weight assessment & counseling (children)	0.576	0.588 (+ 0.012)	0.600 (+ 0.024)	0.612 (+ 0.035)*	0.623 (+ 0.047)*	0.635 (+ 0.059)*	0.647 (+ 0.071)*	0.659 (+ 0.083)*	0.671 (+ 0.095)*	0.683 (+ 0.106)*	0.694 (+ 0.118)*
Weight assessment & counseling (adults)	0.646	0.653 (+ 0.007)	0.659 (+ 0.013)	0.666 (+ 0.020)	0.673 (+ 0.027)	0.680 (+ 0.034)*	0.686 (+ 0.040)*	0.693 (+ 0.047)*	0.700 (+ 0.054)*	0.706 (+ 0.060)*	0.713 (+ 0.067)*
Colorectal cancer screening	0.392	0.399 (+ 0.007)	0.405 (+ 0.013)	0.411 (+ 0.019)	0.417 (+ 0.025)	0.423 (+ 0.031)*	0.430 (+ 0.038)*	0.436 (+ 0.044)*	0.443 (+ 0.051)*	0.449 (+ 0.057)*	0.456 (+ 0.064)*
Childhood immunization status	0.338	0.338 (+ 0.000)	0.338 (+ 0.000)	0.339 (+ 0.001)	0.339 (+ 0.001)	0.339 (+ 0.001)	0.339 (+ 0.001)	0.339 (+ 0.001)	0.340 (+ 0.002)	0.340 (+ 0.002)	0.340 (+ 0.002)
**Chronic disease management**											
Use of appropriate medications for asthma	0.707	0.713 (+ 0.006)	0.719 (+ 0.012)	0.725 (+ 0.018)	0.731 (+ 0.024)	0.737 (+ 0.030)	0.742 (+ 0.035)*	0.748 (+ 0.041)*	0.754 (+ 0.047)*	0.759 (+ 0.052)*	0.765 (+ 0.058)*
Coronary artery disease: lipid therapy	0.723	0.727 (+ 0.004)	0.731 (+ 0.008)	0.735 (+ 0.012)	0.740 (+ 0.017)	0.744 (+ 0.021)	0.748 (+ 0.025)*	0.752 (+ 0.029)*	0.756 (+ 0.033)*	0.760 (+ 0.037)*	0.764 (+ 0.041)*
IVD: use of aspirin or another antithrombotic	0.740	0.745 (+ 0.005)	0.750 (+ 0.010)	0.755 (+ 0.015)	0.759 (+ 0.019)*	0.764 (+ 0.024)*	0.769 (+ 0.029)*	0.774 (+ 0.034)*	0.778 (+ 0.038)*	0.783 (+ 0.043)*	0.788 (+ 0.048)*
Controlling high blood pressure (hypertensive)	0.598	0.602 (+ 0.004)	0.605 (+ 0.007)	0.609 (+ 0.011)	0.613 (+ 0.015)	0.616 (+ 0.018)*	0.620 (+ 0.022)*	0.624 (+ 0.026)*	0.628 (+ 0.030)*	0.631 (+ 0.033)*	0.635 (+ 0.037)*

* *p* < 0.05

Chronic disease measures similarly showed that CHCs improved performance after half of its delivery sites had been designated. The exception to these trends were IVD scores, which began generating improved adherence scores once at least 30% of its sites became designated. The largest change in adherence scores was for the asthma medication adherence. Its mean scores rose from 70.7 to 74.2%, a 3.5 percentage point change, to 76.5%, a 5.8 percentage point change, as the proportion of designated sites increased. Other indicators showed improvements in adherence scores across PCMH saturation groups ranging from a 2.2 percentage point increase in hypertensive adherence scores to a 4.8 percentage point increase among the ischemic vascular disease indicator. Counts of the number of CHCs and delivery sites that were included in each discrete category are shown in Table 6.

**Table 6:** Counts of CHCs and delivery sites by increase in site-level PCMH designation

Proportion of designated sites	No. of CHCs	No. of delivery sites (designated)
0%	262	1135 (0)
1–10%	25	417 (29)
11–20%	68	630 (100)
21–30%	70	645 (173)
31–40%	116	910 (321)
41–50%	172	1062 (501)
51–60%	50	513 (295)
61–70%	72	578 (378)
71–80%	61	433 (327)
81–90%	29	242 (207)
91–100%	114	286 (284)

## Discussion

Medical homes are emerging as the standard for care quality and comprehensive primary care delivery across the country. As of 2018, three quarters of HRSA’s CHCs were designated medical home sites, making HRSA one of the country’s foremost adopters of PCMH-modeled care. Although PCMH transformation has come to represent a “whole-person approach” to primary care delivery, [32, 33] and with numerous examples of improving primary care delivery and lowering overall health care costs, [34–38] there is still an emergent focus on whether it is able to resolve differences in primary care experiences due to racial, socioeconomic, and geographic contexts [39]. Amplifying this limitation are data restrictions that prohibit generating risk-adjusted evaluations of medical home initiatives. These constraints similarly problematize parallel efforts to understand the effectiveness of other initiatives to improve access and quality of primary care [40].

Previous studies have found inconsistent evidence of improved processes and outcomes of care since HRSA began transitioning its CHCs into designated medical homes. One explanation for mixed findings has been the lack of information on the proportion of its delivery sites that are designated medical homes. The Commonwealth Fund Survey, though robust in context, furthers these assumptions through dichotomizing CHCs into a singular classification of designation. What researchers are left with is a binary indicator of PCMH status that does not consider that the vast majority of a CHC’s delivery sites may not be designated. A strength of this study was the ability to use data linkage techniques to measure the association between site-level designation and changes in performance scores. Although site-level data would help answer questions as to the underlying drivers of improved performance under PCMH designation (e.g., access and communication, patient tracking, care management, etc.), in absence of publicly available site-level clinical performance data, the proposed methodology offers an alternative approach to monitoring the association between PCMH designation and changes in care quality using publicly available data.

The study findings add a new perspective as to why using network-level designation reports to infer the extent of site-level PCMH saturation may generate mixed results. Although we found that PCMH designation, on average, remains a positive predicator on clinical performance, the regression models suggest that previous evidence may have been driven by CHCs that designated at least half of its delivery sites. Although our choice to use 10% increments to measure this effect was user-specified, the findings raise new questions pertaining to importance of site-level saturation as well as a potential threshold for when designation leads to better performance. Our findings also provide some initial evidence that once CHCs designate half of their delivery sites they could continue to expect to see a stepwise increase in performance as they approach 100% designation. While many of these increases were in the range of 2 to 3 percentage points, some comparisons showed a 5 to 11 percentage point increase in performance. These findings help to confirm that transformation does in fact matter, but it may be more nuanced then what has previously been reported. These findings are especially important given the inherent challenges that the full overhaul of CHC practice culture requires [41].

At the same time, our findings also suggest a potential ceiling effect of using PCMH status as a universal measure of care quality. If these adherence rates suggest that delivery sites are already working at near-optimum performance (i.e., a clinic’s adherence rate cannot get any higher), then it may become necessary to add risk-adjustment criteria to annual performance reports, much like how the Centers for Medicare and Medicaid Services do for its Hospital Readmissions Reduction Program (HRRP). At the same time, the relatively small improvements in adherence scores across the intervals may also reflect the fact that CHCs have historically emphasized cultural competence, teamwork, and patient-centrism, all of which may confound evaluations of its transformation [42]. The varying effect and differences in magnitude of PCMH designation highlight a need to continue examining their effect more closely, which may require additional site- or patient-level data as well as the ability to risk-adjust performance targets.

One indicator where PCMH designation showed mixed effects was adherence rates for childhood immunization. Designated CHCs had comparably lower benchmark proportions and showed no differences in adherence rates compared to non-designated centers. Whether PCMH recognition is important for increasing adherence to childhood immunization remains a question that is not well answered [43]. However, these trends could also reflect the 2017 changes to HRSA’s immunization indicator, which now includes all patients who have not seen their provider before turning age 2 and has increased the numerator to include Hepatitis A, rotavirus, as well as influenza vaccines. While these changes could explain the decrease in adherence rates from previous years, it does not explain why adherence rates for immunization targets remained significantly lower compared to other measures.

However, these findings should be interpreted within the context of a number of limitations in the data. First, this study represents a point-in-time cross-sectional evaluation of clinical performance derived from CHC performance reports. Although assessments based on cross-sectional data are limited, they remain the most widely available option for monitoring changes in care under the PCMH transition. Attributing differences in quality to differences in the proportion of recognized delivery sites provides one mechanism to address a long-standing limitation within HRSA’s ongoing PCMH evaluations. A related limitation is the lack of longitudinal data to measure improvement over time as well as the lack of data that can be linked back to the delivery site. Although the findings from this study remain novel, they do not replace the benefit that access to site-level clinical reports would bring to these analyses. In absence of fine-scale data from its delivery sites (e.g., continuity of care across sites), the proposed methodology serves as an alternative approach to modeling changes in performance that account for site-level designation.

It is also important to consider these limitations in light of the geocoding and approximate string-matching algorithm. Prior to excluding false positive/negative matches, we were able to confirm 64% of CHCs as having at least one designated delivery site, which was less than the 77% HRSA published for the same year for the lower 48 states and District of Columbia [31]. Three factors could have accounted for the observed differences. One is that we excluded delivery sites whose grantee was accredited through the AAAHC. In 2018, 48 CHCs that oversaw 294 delivery sites had obtained AAAHC accreditation [44]. We excluded the AAAHC database because it uses a network accreditation process rather than site-based designation used by the NCQA and JC. Although the AAAHC grants all delivery sites a specific grace period from which to obtain the similar status as a certified site (e.g., 3 years), it is not possible to determine which of the CHC’s delivery sites are the accredited clinics from its reports. This in some ways perpetuates the limitation embedded in the Commonwealth Survey and HRSA’s PCMH reports that this study sought to overcome. Another factor could be that our data linkage algorithm was too restrictive owing to our emphasis on avoiding false positives. Another is our lack of access to archival data, which required that we link data using different time stamps. Finally, our use of non-PCMH centers as controls required that we exclude recognition time as a potential determinant of care quality, which is a known factor of PCMH performance.

Despite these limitations, the findings from this study come at a precarious time when access to high quality data is urgently needed in order to leverage critical responses to challenges to the impact of COVID-19, particularly within communities that are among the most socially vulnerable. Without access to data that supports the patient-centeredness that PCMH designation brings, we are unlikely to grasp the true significance of COVID-19 on long-term changes in population health outcomes. Harnessing the data linkage and analysis properties of geographic information systems allows researchers to overcome some of the limitations in HRSA’s current data reporting and release statistics and make more robust assessments of patient-centered outcomes for end users. While the lack of site-level data will remain problematic for fully identifying individual strategies that are meeting patient needs, the insight that this methodology affords could make it less difficult to monitor the long-term effect of changes in population health, particularly those that arise as a result of COVID-19.

## Conclusion

One source of variation in previous study findings linking PCMH transformation to improvements in HRSA’s quality reporting has been the lack of information on the proportion of CHC delivery sites that are designated medical homes. A consequence of this limitation is the inability to generate hypotheses of site-level effects on overall clinical performance, which some have argued may be a cause for mixed findings in associations between PCMH designation and performance measures. The absence of this information impedes efforts to evaluate whether the medical home program and policy transitions can be attributed to improvements in patient care. The methodology proposed in this study provides an alternative approach to measuring the association between PCMH status and care quality while also accounting for the proportion of a CHC’s delivery sites that are designated medical homes. The approach has an added benefit in that it is based on publicly available information. Our study findings suggest that transformation does in fact matter, but that it may not appear until a specific proportion of delivery sites become designated. There also appears to be a continued stepwise increase in adherence scores once this threshold is achieved.

## Data Availability

All locational data used to conduct this study is publicly available and able to be mapped through linkages with HRSA facilities stored on its GIS server. A list of currently accredited/recognized NCQA practices can be found at: https://reportcards.ncqa.org/#/practices/list?recognition=Patient-Centered%20Medical%20Home. Joint Commission recognized practices can be found at: https://www.qualitycheck.org/data-download/. AAAHC sites can be found at: https://eweb.aaahc.org/eweb/dynamicpage.aspx?site=aaahc_site&webcode=find_orgs. Readers interested in the SAS code used to build the models should contact the corresponding author.

## Abbreviations

PCMH: Patient-centered medical home
CHC: Community health center
HRSA: Health resources and service administration
NCQA: National COMMITTEE FOR QUALITY ASSURANCE
JC: Joint commission
AAAHC: Accreditation association for ambulatory health care
UDS: Universal data system
